# Probiotics and Postbiotics Derived from Saline/Marine Plant-Based Feedstocks

**DOI:** 10.1007/s12602-025-10617-z

**Published:** 2025-06-17

**Authors:** Stanislav Rudnyckyj, Mette Hedegaard Thomsen

**Affiliations:** Department of Energy, Aalborg University, Esbjerg, Denmark

**Keywords:** Seaweed, Halophytes, Probiotics, Postbiotics, Fermentation, Saline feedstock

## Abstract

The growing demand for the sustainable and cost-effective production of probiotics and postbiotics has highlighted the potential of saline and marine plants as novel substrates. These plants, including seaweeds and halophytes, are abundant and nutrient-rich and require minimal resources, making them ideal candidates for green biorefineries. The incorporation of saline plant-based feedstocks could lower media costs and environmental impact, as these plants do not require arable land or freshwater while contributing to carbon sequestration and sustainable farming. The development of integrated biorefineries could drive economic feasibility by facilitating cost-effective probiotic and postbiotic production. However, challenges such as high salt content and lignocellulosic composition may complicate microbial fermentation. This review examines recent advancements in leveraging naturally salt-tolerant probiotics and efficient bioconversion methods to address these challenges. It explores the nutritional profiles of saline plants, their prebiotic potential, and their synergetic compatibility with diverse probiotic strains, including probiotic bacteria and fungi and their metabolites. Additionally, the review discusses state-of-the-art fermentation techniques tailored to saline plant-based substrates and the possible advantages of saline feedstocks for probiotics and postbiotics production through biorefinery pathways. The work highlights the transformative potential of saline and marine plant-derived probiotics and postbiotics in health supplementation and biotechnological innovation, contributing to biorefinery development within a circular economy framework.

## Introduction

**Fig. 1:**
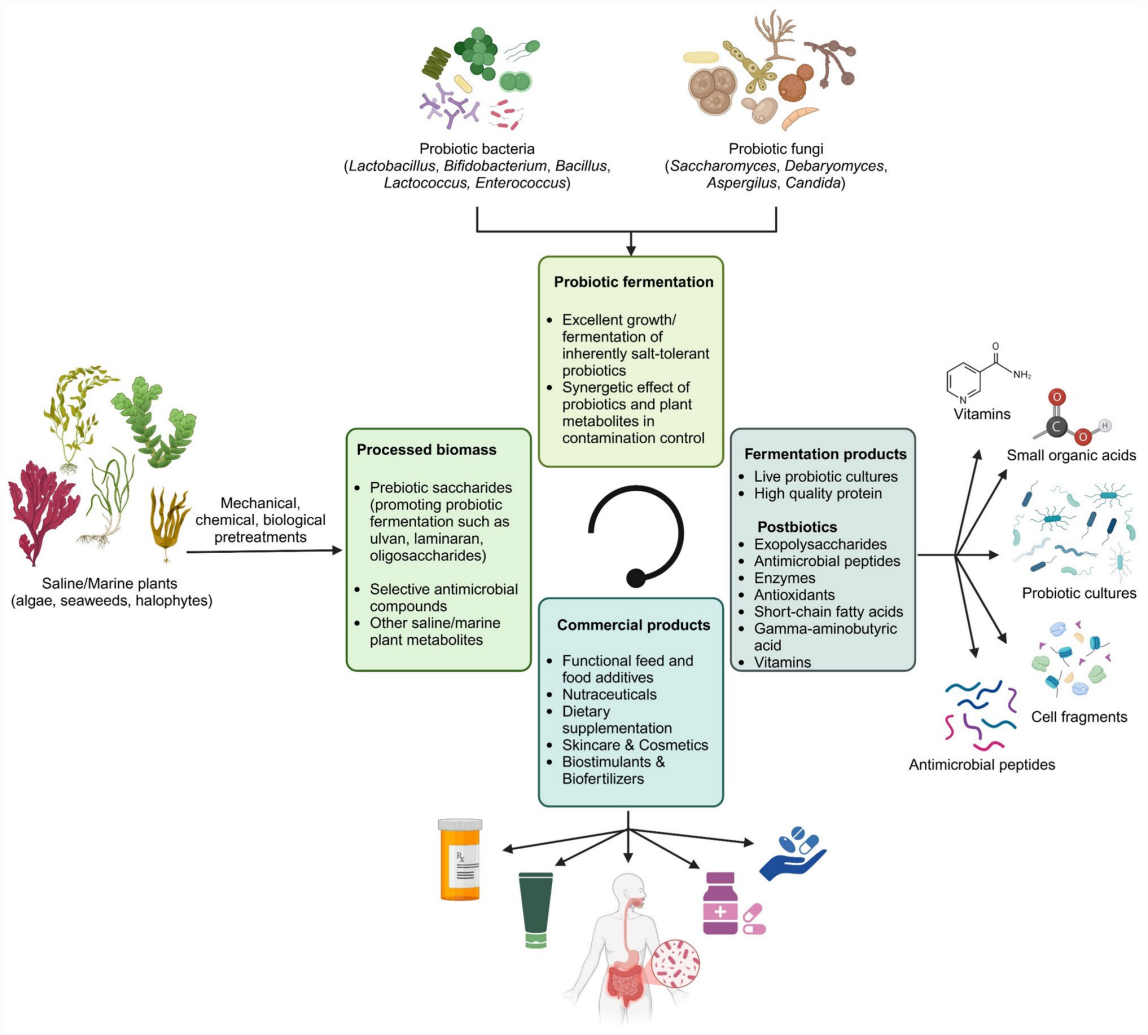
Overview of saline/marine plant-based probiotics and postbiotics (bioactive metabolites), and their commercial applications

The growing field of probiotics, encompassing beneficial microorganisms and their metabolites enhancing human and animal health, necessitates sustainable and cost-effective production methods. Traditional approaches often rely on resource-intensive cultivation methods [1]. With the increasing need for alternative feedstock sources, saline and marine plants offer a unique opportunity due to their abundance, rich nutrient profiles, low to non-resource requirements, and lack of competition with traditional crops [2, 3]. However, the high salt content of these substrates can pose challenges for conventional microbial fermentation, as well as the lignocellulosic nature of plant material [4, 5]. Recent advances in salt-tolerant probiotics and the production of bioactive postbiotics from saline plant matter hold significant promise for applications in green biorefineries, health supplementation, and sustainable/regenerative agriculture. This review explores the potential of utilizing saline/marine plants as novel feedstock for the production of probiotics and postbiotics, addressing current knowledge and future prospects in this emerging field, as illustrated in Fig. 1.

### Types of Probiotics

Probiotics are defined as live microorganisms that provide health benefits to the host [6], and postbiotics, the bioactive compounds produced during fermentation, have gained significant attention for their roles in human health, animal nutrition, and biotechnological applications [7]. Bacterial probiotics constitute a significant domain, including lactic acid bacteria (LAB), which are characterized by the ability to convert sugars into lactic acid. *Lactobacillus*, *Bifidobacterium*, and *Enterococcus* are well-studied, and the most common LAB genera used as probiotics [8]. They were directly connected to the support of gut health and overall maintained human and animal well-being [9–11]. Other less represented but still well-studied and proven bacterial probiotic genera include *Streptococcus*, *Bacillus*, *Escherichia*, *Pediococcus*, *Lactococcus*, and *Propionibacterium* [12–14].

In the case of yeast probiotics, there are significantly fewer strain numbers than bacterial probiotics; however, they are still very relevant. The most commonly used probiotic and clinically proven is *Saccharomyces cerevisiae* var. *boulardii* [15]. This yeast has been extensively studied for its health benefits and safety profile, showing its ability to improve digestion, enhance gut barrier function, and provide antimicrobial activity against pathogens. It is particularly effective in treating antibiotic-associated diarrhea and various gastrointestinal disorders [15]. Moreover, this strain has shown to be an excellent producer of postbiotic compounds, demonstrating the metabolism of a large variety of bioactive compounds [16]. Beyond *S. boulardii*, there are other non-*Saccharomyces* yeasts with probiotic potential, which include genera of *Kluyveromyces*, *Pichia*, *Candida*, *Yarrowia*, *Debaryomyces*, *Kazachstania*, *Wickerhamomyces*, and *Rhodotorula* [17]. Notably, *Kluyveromyces marxianus* is the only non-*Saccharomyces* yeast commercially available probiotic supplement and has extensively been validated for its beneficial effects on human and animal health [18]. Moreover, the discovery of potential probiotic yeast strains continues to grow, driven by increasing research interest in this field. For example, yeast such as *Cyberlindnera jadinii* [19] has shown probiotic potential due to a symbiotic relationship with probiotic bacteria [20] and demonstrated anti-inflammatory effects on the gut [21].

When it comes to filamentous fungi, there were species that demonstrated benefits to animal health and are often derived from traditionally fermented food. The most common genus with potential probiotic properties is *Aspergillus* [22]. Jasim et al. [23] confirmed the probiotic role of *Aspergillus niger* in common carp since it improved growth, immunity, digestion, and fish hematology. *Aspergillus awamori* was demonstrated as an excellent probiotic feed additive to broiler chickens, increasing overall health, metabolism, and growth [24, 25]. *Aspergillus oryzae* is another *Aspergillus* species widely used as a probiotic in poultry, demonstrating benefits such as enhanced growth, improved nutrient digestibility, and better intestinal health [26]. Beyond *Aspergillus*, other less common but notable molds also exhibit probiotic potential. For instance, *Rhizopus oryzae* showed good probiotic properties and fermentative capacities [27]. Similarly, *Rhizopus oligosporus* has been recognized for its probiotic potential, with studies in pigs indicating improved growth, digestion, microbiome balance, and immune function [28].

### Types of Postbiotics

Postbiotics, composed of inactivated/dead microorganisms, their cellular components, and metabolites, can be categorized into various groups based on their chemical origin. Carbohydrates, such as polysaccharides like teichoic acids and galactose-rich polysaccharides, are essential for various biological activities [29]. Exopolysaccharides (EPS), which are high-molecular-weight polymers secreted by microorganisms such as bacteria *Lactobacillus*, *Bifidobacterium*, and *Pseudomonas* and fungi *Kluyveromyces* and *Saccharomyces*, play essential roles in biofilm formation, microbial adhesion, and protection against environmental stressors. EPS have been shown to possess immunomodulatory effects, enhancing the immune response and exhibiting anti-inflammatory properties [30]. Proteins, including specific extracellular peptides such as p40 and p75 molecules, have been shown to exert a significant role in gut health and immune modulation [31]. Additionally, enzymes produced during fermentation processes play a vital role in the breakdown of substrates and can enhance the bioavailability of nutrients, thus mitigating digestive syndromes [32]. Furthermore, certain peptides, known as bacteriocins, act as antimicrobial agents, exhibiting antibacterial or antiviral properties that enhance the host’s immune defense [33].

Short-chain fatty acids (SCFAs) such as acetate, butyrate, and propionate are critical for gut health, serving as energy sources for colonocytes and playing roles in anti-inflammatory processes [34]. Gamma-aminobutyric acid (GABA), a neurotransmitter produced by certain *Lactobacillus* and *Bifidobacterium*, is another important postbiotic that plays a role in reducing stress, improving sleep, and regulating metabolism [35]. Another standard product of bacterial probiotics, lactate, is a significant metabolite that is associated with gut health benefits [36]. Furthermore, organic acids, such as phenyllactic acid (PLA) and propionic acid, play notable roles in modulating gut health and microbial balance [37, 38]. Cell wall fragments derived from bacterial cell walls also contribute to postbiotic activity. These fragments include teichoic acids and lipoteichoic acids, which can stimulate immune responses by activating various signaling pathways in host cells [39]. Another significant group of postbiotics is vitamins, mainly B-group vitamins and vitamin K, produced by both bacterial and fungal probiotics [40, 41].

As research into probiotics and postbiotics continues to evolve, increasing attention is being directed toward the substrates used in their cultivation. The nutritional profile, chemical composition, and bioactive potential of these substrates can significantly influence both microbial growth and the nature of the resulting postbiotic compounds [42, 43]. In this context, saline and marine plants have emerged as promising feedstocks. Their ability to thrive in harsh environments is often accompanied by a unique metabolite profile, including osmoprotectants, phenolics, and polysaccharides, which may synergize with probiotic activity or enhance postbiotic production [44–47]. The following sections explore the composition of these unconventional feedstocks and their compatibility with probiotic cultures.

### Composition of Saline/Marine Plant-Based Feedstocks

Saline and marine plants, including halophytes and seaweeds, possess unique nutritional profiles, potentially supporting probiotic growth. Seaweeds, for instance, are rich in carbohydrates, proteins, lipids, vitamins, and minerals [2, 48]. Their diverse polysaccharide composition, including alginates, fucans, and galactans, offers potential prebiotic effects, stimulating the growth of specific probiotic bacteria [46]. These polysaccharides are known for their beneficial properties, acting as antioxidants, anti-inflammatories, and even anticancer agents [46]. The specific composition varies greatly among species, seasons, and locations [45], necessitating careful selection of seaweed species based on the target probiotic strain and/or economic and environmental needs.

One of the key considerations in biomass utilization is its salt content, which can hinder or even inhibit the pretreatment, cultivation of probiotics, and the production of postbiotics. Salt is often found as part of the ash content, which is typically undesirable in biorefining processes. In some instances, ash may be the predominant component in seaweed biomass, making certain species unsuitable for biorefining. Olsson et al. [2] examined the ash content in various species of red, green, and brown seaweeds along the Swedish west coast, revealing that the ash content ranged between 118 and 419 g/kg of dry weight, depending on the seaweed species. Furthermore, the weight of salt would be considerably higher if the total salt content were considered rather than just metals in the ash. However, taking into account the carbohydrate content of 237 to 557 g/kg dry weight [2], it can be expected that the biomass will be diluted five to ten times with water or aqueous solution for microbial cultivation, ensuring an appropriate range of nutrients. This would result in a metal content range of approximately 5.4 to 31.4 g/L in the cultivation medium, corresponding to 0.5% to 3% w/v, making it a possible feedstock for probiotic cultivation.

Another study of 15 seaweed species of red, green, and brown algae in Sri Lanka demonstrated even greater fluctuations in ash content between species, ranging from 14.1 to 474 g/kg of dry weight [4]. Similarly, a study on brown algae from Danish and Icelandic waters showed that ash content in brown seaweeds ranged from 295 to 544 g/kg of dry weight [49]. Based on these findings, it would be reasonable to consider probiotic strains with high salt tolerance or, ideally, halophilic strains.

As demonstrated by multiple screening studies, seaweeds are generally rich in sugar content, primarily derived from various polysaccharides. A comprehensive review indicates that carbohydrate content ranges from 201 to 767 g/kg dry weight, protein from 23 to 234 g/kg dry weight, and lipids from 30 to 228 g/L [50]. Similarly, these values align with extensive screening conducted by Olsson and colleagues [2], showing a carbohydrate content between 237 and 557 g/kg dry weight and protein levels ranging from 59 to 201 g/kg dry weight. However, these carbohydrates are mostly not available as free fermentable sugars but are instead present as complex polysaccharides with significant structural diversity [51]. For example, green macroalgae primarily contain mannan, ulvan, starch, cellulose, and some monosaccharides (glucose, mannose, rhamnose, xylose), contributing to both structural integrity and energy storage. Red macroalgae are rich in carrageenan, agar, cellulose, lignin, monosaccharides (glucose, galactose), and agarose. Brown macroalgae predominantly contain laminarin, mannitol, alginate, fucoidan, cellulose, and fucose, essential for their flexibility, storage functions, and bioactive properties [51, 52]. It was demonstrated that laminaran stimulated the growth of *Bifidobacteria* and *Bacteroides*, and ulvan stimulated the growth of *Bifidobacteria* and *Lactobacilli*, supporting the idea of prebiotic properties of seaweed saccharides [53].

Although seaweeds are nutritious and can be directly fermented using solid-state fermentation (SSF) or submerged fermentation, the resulting yield and processing time may be inefficient [54]. Therefore, seaweed-based biorefineries often rely on pretreatment methods such as physical, chemical, and enzymatic techniques to recover fermentable nutrients and enhance biomass-to-product conversion efficiency [52, 55].

Emerging super-crops halophytes, adapted to saline environments, also offer unique nutritional profiles. Their high salt tolerance is often coupled with the accumulation of proline and glycine betaine, which might influence probiotic growth [56]. Halophytes are also rich in sugars, proteins, and other essential nutrients [57–59], making them potentially valuable feedstocks. However, the presence of potentially harmful secondary metabolites in some wild halophytes requires careful screening and selection of suitable species [60].

Like seaweeds, halophytes contain a high concentration of salt on a dry matter basis, highlighting their similarity to algal biomass. Ash content in halophytes has been reported to range from 5.2% to 43% w/w of dry weight, varying by species, location, and plant part [61]. Similarly, Hulkko et al. [5] found that ash content in various European halophyte species ranged from 16% to 56% w/w, further emphasizing their compositional resemblance to seaweed biomass. Moreover, halophytes exhibit diverse compositions, with carbohydrate content ranging from 120 to 347 g/kg dry weight, protein content from 150 to 300 g/kg dry weight, and a consistently low lipid content of less than 4% w/w on a dry matter basis [5]. Abideen and coworkers [62] analyzed the polysaccharide composition of cellulose and hemicellulose in dozens of halophyte species, reporting values between 220 and 667 g/kg dry weight.

Additionally, both seaweeds and halophytes are rich in bioactive compounds such as chlorophylls, carotenoids, phenolics, and vitamins [44, 57, 63]. It has been shown that seaweed metabolites are frequently prebiotic and can modulate microbial communities, promoting the growth of beneficial probiotic bacteria [64–66]. Likewise, halophyte extracts have been shown to stimulate the growth of probiotic strains while selectively inhibiting common pathogens [67, 68]. Moreover, halophyte-based extracts have been demonstrated as excellent antimicrobial agents against common pathogens [69, 70]. For instance, Campana et al. [71] conducted a comparative analysis of essential oils from various halophyte species and found that *Cuminum cyminum*, *Crithmum maritimum*, and *Pimpinella anisum* were effective against all tested microorganisms, including *Escherichia coli*, *Listeria monocytogenes*, *Staphylococcus aureus*, *Pseudomonas fluorescens*, and *Candida albicans*. Essaidi and colleagues [72] also reported the antimicrobial activity of *Salicornia herbacea* extract against several pathogenic strains, such as *S. aureus*, *E. coli*, and *Klebsiella pneumoniae*. In another study, *Avicennia marina* extract showed strong inhibition against *C. albicans* and *B. subtilis*, with moderate effects on *Salmonella typhimurium* and *Vibrio damsela* [73]. Additionally, Sánchez-Hernández et al. [74] demonstrated that extracts from *Limonium binervosum* possess both antimicrobial and antifungal activity, partially or completely suppressing the growth of plant pathogens including *Xylophilus ampelinus*, *Erwinia amylovora*, and *Diplodia seriata*. These findings support the idea that the selective antimicrobial compounds naturally found in saline/marine plants, which target non-probiotic microorganisms, could help control contamination during fermentation and extend product shelf life, thereby significantly enhancing the cost-effectiveness of the biorefinery concept. The potential of selective antimicrobial compounds naturally present in saline and marine plants to control contamination during fermentation, enhance product shelf life, and ultimately improve the cost-effectiveness of the biorefinery concept.

In the case of halophyte-based biorefinery, preference lies in the extraction of high-value bioactive compounds from juiced fibers, as demonstrated by Fredsgaard et al. [75] and Hulkko et al. [59]. Extract-free halophyte lignocellulosic fibers can be used to produce bulk biochemicals [76–78] and bioenergy [79], and halophyte juice can be processed in protein-enriched feed [80]. Generally, the extraction and processing of bioactive compounds from saline/marine plants require efficient and sustainable methods. Biorefinery approaches, which integrate multiple processing steps to maximize resource efficiency, are particularly relevant in this scenario [52, 55, 81]. The integration of bioconversion processes further enhances the sustainability of this approach. Microbial fermentations, using specific bacteria and/or fungi, can convert plant biomass into valuable products, including probiotics themselves [54, 82, 83]. This approach could minimize waste and enhance the overall efficiency of the process. Furthermore, the use of microbial fermentations can also improve the digestibility and bioavailability of nutrients in the seaweed and halophyte biomasses [84, 85], contributing to a circular economy.

### Compatibility of Probiotic Cultures with Saline Environments and Plant Metabolites

The selection of appropriate probiotic strains is crucial for ensuring their effectiveness in production from saline feedstocks. Different strains exhibit varying levels of tolerance to salinity and the specific components found in seaweed and halophytes, which can influence their viability and performance. Interestingly, probiotic microorganisms are inherently tolerant to high salt concentrations, making them ideal candidates for production. The reason for their tolerance lies in their ability to adapt cellular mechanisms, such as osmotic regulation and stress response pathways, which allow them to thrive in environments with elevated salinity [86, 87]. This tolerance enhances their potential for use in the fermentation of products derived from saline sources, improving both their stability and efficacy in such applications.

Probiotic bacterial genera, including *Lactobacillus*, *Bifidobacterium*, and *Bacillus*, are known for their salt tolerance, as demonstrated by numerous studies. For instance, *Lactobacillus sakei* has been shown to withstand up to 10% NaCl (w/v), while *Lactobacillus oris* tolerates up to 7% NaCl [88, 89]. Additionally, *Lactobacillus* strains, like *L. plantarum*, *L. fermentum*, and *L. paracasei*, demonstrate survival in environments containing up to 4% NaCl (w/v) [90].

*Bifidobacterium* strains are also known to tolerate up to 10% NaCl [91], with some studies even reporting survival under more extreme salinity levels. Borges et al. [92] highlighted the impressive survival capabilities of probiotic strains such as *Lactobacillus casei*, *L. paracasei*, *L. acidophilus*, and *Bifidobacterium animalis*, all of which withstood up to 25% (w/v) NaCl. Among the *Bacillus* species, *Bacillus subtilis* is another strain that shows remarkable resistance, enduring up to 10% NaCl (w/v) [93]. Furthermore, research by Yan and colleagues [94] demonstrated that *Bacillus coagulans* could resist concentrations of NaCl up to 0.8 mol/L, which is approximately equivalent to 4.7% (w/v) NaCl. Other probiotic strains, such as *Lactococcus lactis*, can generally endure up to 5% (w/v) NaCl [95], and some strains have even shown resilience to concentrations as high as 15% (w/v) NaCl [96].

Similar to probiotic bacteria, probiotic yeast strains also exhibit remarkable salt tolerance. For instance, Sengun et al. [97] demonstrated that various probiotic strains of *Pichia* and *Saccharomyces* were still viable at 10% (w/v) NaCl. Additionally, multiple strains of *Debaryomyces hansenii* and *Torulaspora delbrueckii* showed growth at 10% (w/v) NaCl and beyond [98]. Reyes-Becerril and coworkers [99] also presented probiotic marine yeast *Y. lipolytica* strains that grow at 6.5% (w/v) NaCl. These findings suggest that probiotic or potentially probiotic yeasts are well-suited for green biorefinery applications, particularly in the conversion of salty biomass into microbial cells and/or their metabolic products.

Furthermore, as indicated in the previous section, probiotic microorganisms exhibit an affinity for plant bioactive compounds, such as polyphenols, leading to a dual effect. On the one hand, these compounds promote the growth and colonization of probiotic bacteria, while on the other, they help reduce the presence of pathogenic bacteria [100–102]. The literature on the specific effects of polyphenols and other bioactive compounds from seaweed and halophytes on probiotic strains remains relatively limited compared to other plant sources. However, multiple studies have documented the impact of algal metabolites on probiotic microorganisms. For example, in an 8-week pilot study, mice supplemented with 0.04% w/w astaxanthin, a common algal antioxidant, showed an increased population of *Bifidobacterium* [103]. Charoensiddhi et al. [104] demonstrated that polysaccharide and phlorotannin-enriched extracts of the brown seaweed *Ecklonia radiata* positively influence the growth of *Bifidobacterium*, *Lactobacillus*, and *Clostridium coccoides*. For halophyte extracts, only one study has directly linked *Crithmum maritimum* L. extract to the growth stimulation of *Lactobacillus bulgaricus* [68]. This extract also demonstrated antimicrobial activity against the pathogens *S. aureus*, *Staphylococcus epidermidis*, *Candida albicans*, and *Candida parapsilosis*, supporting the idea of the dual effect of the halophyte-based bioactive compounds [68].

## Probiotic and Postbiotic Products Derived from Saline/Marine Plant-Based Feedstocks

**Fig. 2:**
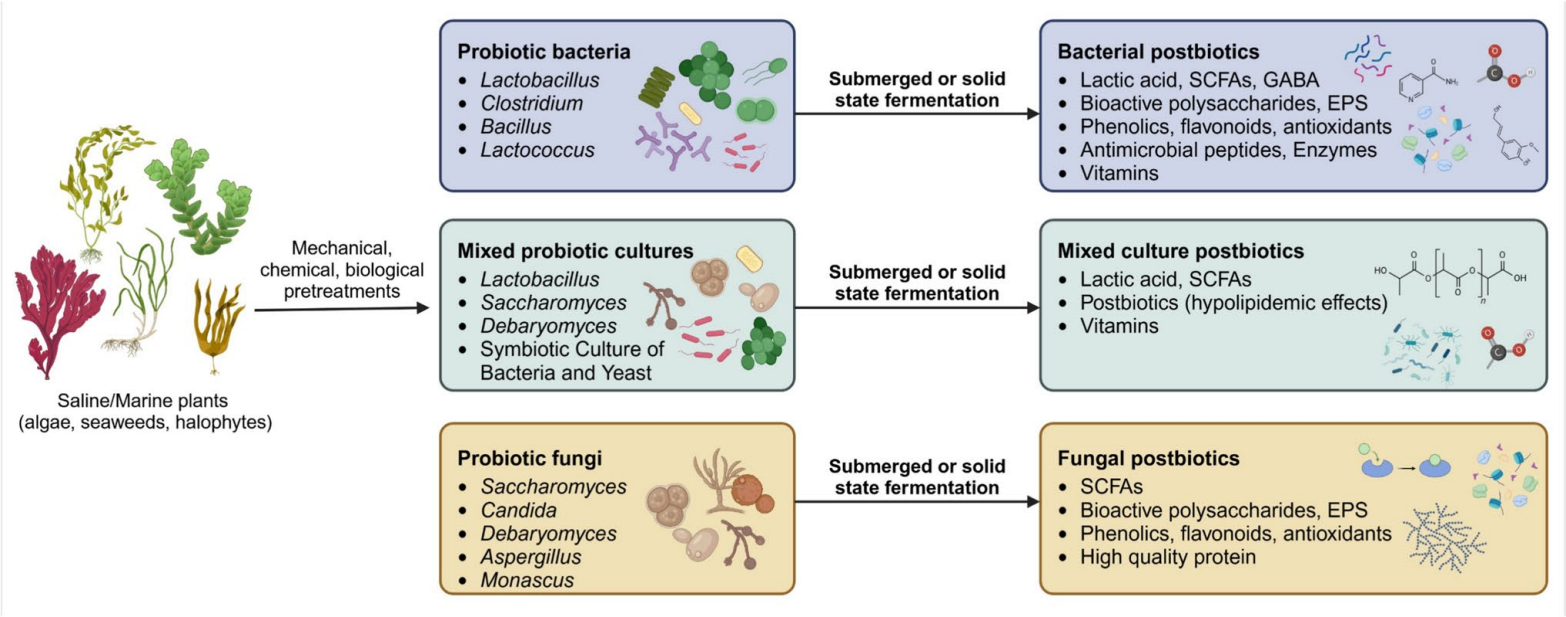
Overview of previously cultivated microbial genera on saline/marine plants and their postbiotic products

Probiotically fermented seaweed has long been a part of various traditional cuisines. For instance, in Japan, fermented kelp is known as Kombu Tsukudani, while in Ireland, Dillisk (*Palmaria palmata*) has historically been fermented. In Korea, seaweed is commonly incorporated into fermented dishes such as kimchi. Traditionally, fermentation was used to enhance flavor and preserve food. However, in the present day, fermentation of seaweed and other saline plants is not only viewed as a method of food production but also as a means to generate specific bioactive compounds, such as postbiotics and beneficial microbial cultures with industrial applications, as presented in Fig. 2.

### Bacterial Probiotics and Postbiotics Derived from Macroalgae-Based Feedstocks

The most extensively studied cultures belong to the *Lactobacillus* genus, demonstrating promising cell biomass and metabolite yields when cultivated on algal-based biomass, as demonstrated in Table 1. Nagarajan et al. [105] demonstrated an impressive conversion of *Ulva* sp., *Gracilaria* sp., and *Sargassum cristaefolium* hydrolysates using multiple *Lactobacillus* strains, achieving over 90% sugar-to-product conversion into organic acids, yielding up to 37.6 g/L in broth, along with microbial cultures. Similarly, Lin et al. [110] showed excellent production of *L. acidophilus* BCRC 10695 and *L. plantarum* BCRC 12327 on *Gracilaria* sp. hydrolysate, reaching 9.23 LAB count (log CFU/mL) and lactic acid concentration of 19.32 g/L. *L. plantarum* and *L. brevis* are particularly interesting producers, as demonstrated by multiple studies. For example, *L. plantarum* MTCC 1407 achieved a lactic acid yield of 109 g/L when fermenting hydrolysates of *Kappaphycus alvarezii* and *Gracilaria corticate* [106]. In another study, *L. plantarum* DSM 20174 fermented *Ulva* spp. hydrolysate, producing 0.9 g of lactic acid per gram of monomeric sugar consumed [126]. Furthermore, *L. plantarum* was compared to other *Lactobacillus* strains in a separate study, showing the highest cell mass production along with organic acid generation when utilizing *Enteromorpha prolifera* hydrolysate [115]. Meanwhile, *L. brevis* has shown the ability to efficiently metabolize mannitol, a common sugar derivative in brown algae, converting it into GABA at concentrations exceeding 3 g/L while simultaneously producing viable probiotic cells [119], making it a promising microbial cell factory. Furthermore, consortia of *L. plantarum* and *L. brevis* have demonstrated strong performance on seaweed hydrolysates, achieving cell densities over 2.7×10^7^ CFU/mL alongside acetic and lactic acid production [108]. These findings underscore the potential of *Lactobacillus* strains in the bioconversion of marine biomass, not only for organic acid production but also for the development of functional probiotic applications. Another bacterium worth considering is *Clostridium acetobutylicum*, which has shown robust growth and production on seaweed-based media, as shown in Table 1. However, its bioconversion processes are generally time-intensive, often requiring days to weeks of cultivation.

**Table 1:** Bacterial probiotics and postbiotics production from specific seaweed species

Type of algae	Probiotic strain	Product	Cultivation conditions	Reference
*Ulva* sp., *Gracilaria* sp., *Sargassum cristaefolium*	*L. plantarum*	L-Lactic acid. acetic acid, probiotics	Fermentation in liquid phase at pH 5.5, 30 °C, 200 RPM	[105]
	*L. sakei*			
	*L. rhamnosus*			
	*W. cibaria*			
	*W.* sp.			
	*W. paramesenteroides*			
*Ulva fasciata*, *Gracilaria corticata*, and *Kappaphycus alvarezii*	*L. plantarum* MTCC 1407	Lactic acid	Fermentation in liquid phase at 37 °C, 100 RPM for 7 days	[106]
	*L. plantarum* MTCC 6161			
*Alaria esculenta* and *Saccharina latissima*	*L. plantarum* ATCC 8014	SCFAs (postbiotics)	Fermentation in liquid phase at 37 °C, 100 RPM for 24 h	[107]
*A. esculenta*	Consortia of three *L. plantarum* and one *L. brevis*	Probiotics, lactic acid, acetic acid	Wet fermentation at 37 °C, 100 RPM for 7 days	[108]
*Gracilaria gracilis*	*L. acidophilus*	Postbiotics, antioxidants, enzymes	Wet fermentation at 30 °C and 37 °C	[109]
	*L. sakei*, *Staphylococcus carnosus*, and *Staphylococcus xylosus*			
	*Staphylococcus xylosus*			
*Gracilaria* sp.	*L. acidophilus* BCRC 10695 and *L. plantarum* BCRC 12327	Probiotics and postbiotics	Fermentation in liquid phase at 30 °C for 72 h	[110]
*Saccharina japonica*	*L. brevis* KCL010	Prebiotics and GABA	Fermentation in liquid phase at 30 °C, 150 RPM for 120 h	[111]
*Bangia fusco-purpurea*	*L. delbrueckii CICC 6045*	Probiotics and postbiotics	Fermentation in liquid phase at 37 °C for 48 h	[112]
	*L. plantarum CICC 6076*			
*Ecklonia cava*	*L. brevis*	Bioactive polysaccharides	Fermentation in liquid phase at 30 °C, 1200 RPM for 24 h	[113]
*E. cava*	*L. brevis*	Bioactive polysaccharides	Fermentation in liquid phase at 30 °C, 1200 RPM for 24 h	[114]
*Enteromorpha prolifera*	*L. brevis KCTC 3498*	Probiotics and organic acids	Fermentation in liquid phase at 30 to 37 °C, 170 RPM for 48 h	[115]
	*L. casei* KCTC 3260			
	*L. plantarum* KACC 11451			
	*L. rhamnosus* KCTC 3237			
	*L. salivarius* KACC 10006			
*Eucheuma cottonii*	*B. coagulans ATCC 7050*	Lactic acid	Fermentation in liquid phase at 37 °C, 100 RPM for 24 h	[116]
	*L. acidophilus-14*			
*Laminaria japonica*	*B. subtilis* N2	Probiotics and anti-inflammatory agents	Fermentation in liquid phase	[117]
*Porphyra*	*L. plantarum* KP3	Lactic acid	Fermentation in liquid phase at 37 °C for 120 h	[118]
	*L. plantarum* KP4			
	*Leuconostoc mesenteroides* K8			
	*L. paracasei* subsp. *paracasei* DP2			
*Undaria pinnatifida*	*L. brevis* KCL010	GABA and probiotics	Fermentation in liquid phase at 30 °C, 150 RPM for 72 h	[119]
*Ulva fasciata*	*Lactococcus lactis*	Probiotics and organic acids	Wet fermentation at 30 °C for two days	[120]
*Gelidium amansii*	*C. acetobutylicum* KCTC 1790	Probiotics, acetic acid, butyric acid, antioxidants	Fermentation in liquid phase at 37 °C, 150 RPM for 7 days	[121]
*Gelidium amansii*	*C. acetobutylicum* KCTC 1790	Butyric acid	Fermentation in liquid phase at 37 °C, 150 RPM for 9 days	[122]
*S. japonica*	*C. acetobutylicum* KCTC 1790	Probiotics, acetic acid, butyric acid	Fermentation in liquid phase at 37 °C, 150 RPM for 13 days	[123]
	*C. tyrobutyricum* KCTC 5387			
*S. japonica* and *U. pinnatifida*	*C. tyrobutyricum ATCC 25755*	Probiotics, acetic acid, butyric acid	Fermentation in liquid phase at 37 °C, 150 RPM	[124]
*Saccharina* spp.	*C. acetobutylicum* ATCC 824	Probiotics, acetic acid, butyric acid	Fermentation in liquid phase at 37 °C	[125]

### Fungal Probiotics and Postbiotics Derived from Macroalgae-Based Feedstocks

In the case of fungal probiotics derived from seaweeds, the yeasts *S. cerevisiae* and *Candida utilis* (also known as *C. jadinii*) are the most prominent, while the filamentous fungus *A. oryzae* is also noteworthy, as shown in Table 2. However, fungal probiotics are significantly less represented and studied than bacterial cultures. *S. cerevisiae* demonstrated organic acid production of 55.8 g/L, comprising lactic, acetic, and tartaric acids, when cultivated on *A. esculenta*-based feedstock, and 51.5 g/L on *S. latissimi*-based feedstock, confirming its suitability for postbiotic acid production [107]. Additionally, *S. cerevisiae* exhibited comparable cell growth on *K. alvarezii*-based media and synthetic potato dextrose broth, reaching over 8.5 log CFU/mL [135]. For *C. utilis*, studies have reported its ability to grow and produce antimicrobial compounds in *Ecklonia bicyclis* water extracts, effectively inhibiting pathogenic growth [130]. More broadly, research has focused on its potential to release anti-inflammatory compounds, such as phenolics, from algal biomass [128, 129]. Furthermore, both yeasts demonstrate superior nutritional content, with highly digestible protein, making them attractive candidates for biorefinery applications [139]. As for *A. oryzae*, it is a well-studied GRAS (Generally Recognized as Safe) species, traditionally used in Asian food fermentation. It has been successfully cultivated on *K. alvarezii* [134], *S. japonica* [137], and *P. palmata* [138], highlighting its potential for fungal-based bioprocessing of marine biomass. Another particularly interesting mold is *Monascus* spp., widely used in East Asian food fermentation, known for its ability to bioconvert plant biomass into postbiotics such as phenolic compounds and antioxidants [131, 132].

**Table 2:** Fungal probiotics and postbiotics production from specific seaweed species

Type of algae	Probiotic strain	Product	Cultivation conditions	Reference
*A. esculenta* and *S. latissima*	*S. cerevisiae* MTCC 180	SCFAs (postbiotics)	Fermentation in liquid phase at 30 °C, 100 RPM for 24 h	[107]
*Ecklonia cava*	*S. cerevisiae*	Bioactive polysaccharides	Fermentation in liquid phase at 30 °C, 1200 RPM for 24 h	[113]
	*C. utilis*			
*Cystoseira trinodis*	*A. niger* Tiegh.	Antioxidants (postbiotics)	Wet fermentation at 28 °C, 120 RPM for 3 days	[127]
	*Dendryphiella arenaria* Nicot			
	*Aspergillus chevalieri* L. Mangin			
	*Chaetomium funicola* Cooke			
	*Stachybotrys chartarum* (Ehrenb.) S. Hughes			
	*Aspergillus nidulans* (Eidam) Vuill.			
*E. cava*	*C. utilis* ATCC 9950	Phlorotannin (anti-inflammatory)	Fermentation in liquid phase at 30 °C, 120 RPM for 24 h	[128]
*E. cava*	*C. utilis*	Phenolics/antioxidants (postbiotics)	Fermentation in liquid phase at 30 °C, 120 RPM for 24 h	[129]
*E. cava*	*S. cerevisiae*	Bioactive polysaccharides	Fermentation in liquid phase at 30 °C, 1200 RPM for 24 h	[114]
	*C. utilis*			
*Eisenia bicyclis*	*C. utilis YM-1*	Antimicrobial activity (postbiotics)	Fermentation in liquid phase at 30 °C, 120 RPM for 24 h	[130]
*S. japonica*	Red yeast rice (*Monascus purpureus*)	Phenolics, flavonoids, and antioxidants (postbiotic)	Solid stare fermentation	[131]
*S. japonica*	*Monascus purpureus* KCCM 60168	Phenolics, flavonoids, and antioxidants (postbiotic)	Wet fermentation with 50% moisture content at 30 °C, 20 days	[132]
	*Monascus kaoliang* KCCM 60154			
*Undaria pinnatifida*	*Monascus purpureus* KCCM 60168	Phenolics, flavonoids, and antioxidants (postbiotic)	Wet fermentation with 50% moisture content at 30 °C, 20 days	[132]
	*Monascus kaoliang* KCCM 60154			
*Durvillaea* spp.	Co-culture of *Pleurotus*, *Lentinula*, *Hericium*, and *Ganoderma*	Mycoprotein, amino acids, antioxidants (postbiotics)	Fermentation in liquid phase	[133]
*K. alvarezii*	*A. oryzae*	Phenolics, flavonoids, and antioxidants (postbiotic)	Solid-state fermentation with 70% moisture content at 30 °C for 2 to 6 days	[134]
*K. alvarezii*	*S. cerevisiae*	Probiotic feed additive	Fermentation in liquid phase at 28 °C, 125 RPM for 48 h	[135]
*Macrocystis pyrifera*	*Paradendryphiella salina* 100654	Mycoprotein, phenolics, antioxidants (postbiotics)	Fermentation in liquid phase at 25 °C, 200 RPM for 8 days	[85]
*Ulva* spp.	*Paradendryphiella salina* 100654	Mycoproteins as feed additive	Fermentation in liquid phase at 25 °C, 200 RPM for 4 days	[136]
*S. japonica*	*A. oryzae*	Phenolics, flavonoids, and antioxidants (postbiotic)	Fermentation in liquid phase at 25 °C for 7 days	[137]
*Palmaria palmata*	*Rhizopusmicroscopus* var. *chinensis* IHEM No. 6048	Protein	Wet fermentation at 37 °C for 6 days	[138]
	*A. oryzae NRRL 1988*			
	*Trichoderma pseudokoningii*		Wet fermentation at 37 °C for 14 days	

### Mixed Culture Probiotics and Postbiotics Derived from Macroalgae-Based Feedstocks

Several studies have explored co-cultures of fungal and bacterial strains, as summarized in Table 3, with some achieving higher yields of target products. For instance, Kombucha SCOBY (Symbiotic Culture of Bacteria and Yeast) cultivated on *A. esculenta* and *S. latissima*-based feedstocks demonstrated enhanced production of organic acids, including lactic, tartaric, and acetic acids, compared to pure cultures of *S. cerevisiae* and *L. plantarum*. Specifically, mixed culture SCOBY fermentation produced 56 g/L of total organic acids from *S. latissima* hydrolysate and 63 g/L from *A. esculenta* hydrolysate, emphasizing the potential of microbial consortia to improve bioconversion efficiency [107].

**Table 3:** Mixed bacterial and yeast cultures for probiotics and postbiotics production from specific seaweed species

Type of algae	Probiotic strain	Product	Cultivation conditions	Reference
*A. esculenta* and *S. latissima*	Kombucha SCOBY (Symbiotic Culture of Bacteria and Yeast)	SCFAs (postbiotics)	Wet fermentation at 25 °C, 50 % relative humidity, 14 days	[107]
*Gracilaria vermiculophylla*	*L. casei* B5201*, D. hansenii* Y5201, and *Candida* sp. Y5206	Lactic acid	Fermentation in liquid phase	[140]
*Laminaria japonica*	*S. cerevisiae* AMnb091 *and L. plantarum* LP1406	Postbiotics (hypolipidemic effects)	Fermentation in liquid phase at 30 °C for 2 days with shaking (180 r/min)	[141]

### Probiotics and Postbiotics Derived from Halophyte-Based Media

Less explored but emerging as a promising second-generation feedstock, halophytes have shown high potential for probiotic growth and metabolite production. Multiple studies have demonstrated their effectiveness as fermentation substrates. For instance, *Salicornia ramosissima* has been successfully used as a salt substitute in white cabbage fermentation, resulting in increased antioxidant activity and total phenol content compared to the control [142]. In another study, Maoloni et al. [143] demonstrated that *L. plantarum* IMC 509, along with a combination of *L. rhamnosus* IMC 501® and *L. paracasei* IMC 502®, were able to survive and maintain their viability for 44 days when incubated in brined Sea Fennel (*Crithmum maritimum* L.), highlighting the potential of halophytes as sustainable substrates for probiotics. Additionally, Hulkko [144] demonstrated the successful cultivation of *L. plantarum* and *L. salivarius* on the green juice of *S. ramosissima* and *Tripolium pannonicum*, resulting in the production of lactic and acetic acids, suggesting that halophyte juices are suitable for *Lactobacillus* cultivation. Similarly, it was found that *S. cerevisiae* effectively ferments on halophyte-based hydrolysates, including *Juncus maritimus* [145], *Salicornia sinus-persica* [83], and *Salicornia bigelovii* [76, 146], further supporting the use of halophytes as viable fermentation substrates. Moreover, Alassali et al. [83] demonstrated that *S. cerevisiae* exhibited excellent growth on *S. sinus-persica* fresh juice, accompanied by the production of acetic and lactic acids.

## Economic, Environmental, and Industrial Perspectives

The global probiotic market is currently valued at approximately 73 billion USD and is projected to grow to 85 billion USD by 2027 [147]. In contrast, postbiotics do not have a separate market category. Instead, they are generally incorporated into nutraceuticals. The nutraceutical industry had an estimated global market cap of 591 billion USD in 2024 and is forecasted to grow at a compound annual growth rate (CAGR) of approximately 7.6% from 2025 to 2030 [148]. This growth is driven by increasing consumer health awareness and the demand for more “traditional” or “natural” alternatives [149, 150].

In regard to economic and environmental considerations, economic analysis of traditional probiotic production, particularly LAB, has revealed that media costs account for over 40% of total production expenses, suggesting an alternative carbon source could considerably benefit the overall cost-effectiveness of the production [1]. Utilizing saline and marine plants as feedstocks for probiotic production presents several environmental advantages. Unlike conventional agricultural crops, seaweed and halophyte cultivation do not compete for arable land or freshwater resources [3], thereby reducing the environmental footprint. Additionally, seaweed and halophytes contribute to carbon sequestration, aiding in climate change mitigation [48, 151]. Halophyte cultivation on saline lands could also prevent land degradation and help restore salinized soils and degraded ecosystems [60].

From the circular economy perspective, the feasibility of using saline and marine plants depends on multiple factors, including harvesting, processing, and cultivation costs. The natural abundance and accessibility of these plants, especially in coastal regions, may help lower production costs [3]. Techno-economic analyses indicate that establishing integrated facilities near the shore could significantly reduce operational costs [152]. Furthermore, the production of value-added byproducts such as plant extracts and bioactive compounds could further enhance economic viability.

### Industrial Prospects

In saline-based biorefineries, high-value product generation is the primary driver for industrial adoption, ensuring economic feasibility [153]. For example, the EU-funded Macro Cascade project evaluated the scalability, feasibility, and profitability of various seaweed-based biorefinery products, including probiotic feed and food, algal-derived saccharides (mannitol, laminarin, fucoidan, alginate), and prebiotic oligosaccharides. The study concluded that probiotic feed and food offered the most favorable economic outcomes among all investigated scenarios [154].

Another study by Nazemi et al. [155] investigated the techno-economic aspects of various process approaches using brown macroalgae as feedstock. The analysis demonstrated that producing biofuels was not economically viable. Instead, only high-value chemicals derived from native algal metabolites showed positive economic outcomes, generating approximately 374$/tonne of dried algae biomass, assuming a plant capacity of 500 ktonne/year. Similarly, a separate study on a biorefinery based on *S. latissima* found that the most profitable outcomes were achieved through the production of value-added products such as alginate, mannitol, protein, laminarin, and fertilizer [156]. The best scenario resulted in an estimated return of 506$/tonne of dried algae biomass at a plant capacity of 200 ktonne/year. In the case of halophytes, there is limited research on the economic feasibility of halophyte-based biorefineries. Nevertheless, a techno-economic assessment of *Salicornia* sp. as a feedstock for jet fuel production, using *Hermetia illucens* for sugar-to-lipid conversion, demonstrated that the process can be both feasible and profitable [81]. However, in the context of nutraceuticals, particularly probiotics and postbiotics, techno-economic evaluations are still lacking and remain to be thoroughly investigated. Additionally, the use of plant-based feedstocks could eliminate traditional unit operations, such as cell separation and washing, thereby reducing both capital expenditures (CAPEX) and operational expenditures (OPEX) [157].

However, several challenges must be considered for the successful implementation of saline/marine plant-based biorefineries. These include:

1. High salt content, which may cause equipment corrosion to some degree [158]
2. Seasonal and climate-dependent variations, as the chemical composition and growth of seaweeds change based on season and location [159]
3. Pretreatment requirements, which may necessitate enzyme and/or chemical applications to optimize biomass utilization [51, 52]
4. Scaling up bioprocesses, as laboratory-scale successes may not directly translate to industrial settings due to process inefficiencies, contamination risks, or inconsistent yields
5. Energy and resource intensity, since processing plant biomass may require high energy inputs, particularly for drying, extraction, and fractionation, increasing operational costs
6. Storage and shelf life, as saline/marine-derived probiotic products may have stability challenges, requiring specialized storage conditions to maintain viability.
7. Market acceptance and consumer perception, where despite sustainability benefits, consumer skepticism about marine/saline plant-derived probiotics and postbiotics could affect demand, necessitating educational efforts
8. Biodiversity and ecosystem concerns, since large-scale harvesting of marine/saline plants might have unintended environmental consequences, including biodiversity loss and habitat disruption
9. Infrastructure and investment gaps, as specialized bioreactors, corrosion-resistant equipment, and coastal facilities require significant initial investments
10. Regulatory barriers, where novel production methods using marine/saline plants may face additional scrutiny from food safety and pharmaceutical regulatory bodies, delaying approvals

### Strain Improvement and Genetic Engineering Considerations

One innovative strategy to overcome process limitations in saline/marine plant-based biorefineries is the genetic improvement of microbial strains. Strain improvement can enhance salt tolerance in probiotic microbes, enabling better growth and metabolic activity under high-salinity conditions typical of halophyte or marine biomass. These modifications may also strengthen their ability to break down complex polysaccharides such as lignocellulose and to withstand or metabolize inhibitory compounds formed during biomass pretreatment, ultimately leading to more efficient bioconversion processes [160]. Generally, direct genetic modifications in probiotic cultures are explored to enhance or introduce specific therapeutic traits, thereby increasing the product’s value. Such genetically modified probiotics (GMPs) are often considered therapeutic agents rather than standard nutraceuticals [161, 162]. For example, *L. lactis* IL1403 was engineered to secrete antimicrobial peptides such as alyteserin and A3 APO, effectively inhibiting *Salmonella* and *E. coli* without compromising host viability, demonstrating its potential as an antibiotic alternative [163]. Another *L. lactis* strain and *L. casei* were engineered to produce elafin, a natural protease inhibitor, for potential treatment of inflammatory bowel disease in humans [164].

However, it is important to note that the use of genetically modified microorganisms (GMOs) in food and feed additives, categories that include probiotics and postbiotics, is subject to strict regulatory restrictions [165]. Due to these regulatory constraints, probiotic strain improvement efforts have primarily focused on non-GMO methods such as adaptive laboratory evolution (ALE) based on natural selection. For instance, Papadopoulou et al. [166] demonstrated that LAB originally isolated from seaweed, when subjected to ALE under saline conditions, developed enhanced salt tolerance and lactic acid production compared to their wild-type ancestors. By the end of the evolutionary period, *L. plantarum* and *Enterococcus faecium* exhibited improved salt tolerance, with resistance increasing by 1.29-fold and 1.75-fold, respectively, enabling growth in media containing over 71 g/L NaCl. Similarly, Han and colleagues [167] showed that ALE improved *L. plantarum* strains to tolerate up to 10% (w/v) NaCl, with some evolved isolates exhibiting comparable growth at 8% (w/v) NaCl to that observed in media without extra salt. In another study, *S. cerevisiae* was adapted for increased salt tolerance via ALE [168].

In the case of plant-derived sugars, many probiotic strains naturally possess enzymes capable of degrading plant polysaccharides. For example, *B. subtilis* AMS6, isolated from traditionally fermented soybeans, exhibited cellulolytic activity [169], while *L. plantarum* RI11, from Malaysian food, was shown to produce extracellular cellulolytic and hemicellulolytic enzymes [170]. These capabilities can be particularly observed in mixed microbial systems such as the gut microbiome, which is well known for fiber “plant polysaccharide” degradation [171]. To further enhance performance, ALE has been used to evolve *Pediococcus acidilactici* ZY15 for improved utilization of lignocellulose-derived sugars and inhibitory compounds formed during biomass pretreatment [172]. Additionally, combining ALE with direct metabolic engineering enabled the development of *P. acidilactici* ZB220, capable of efficiently co-utilizing lignocellulose-derived sugars, leading to increased product yields [173, 174].

Strain improvement can also boost microbial tolerance to inhibitors generated during biomass pretreatment, such as furfural, HMF, acetic acid, and phenolics. These compounds often impair microbial growth and fermentation [175]. For example, *C. tyrobutyricum* was engineered for furfural resistance, resulting in increased butyrate production [176]. Similarly, genetically modified *P. acidilactici* showed significantly improved tolerance to several common inhibitors, including vanillin, syringaldehyde, and HMF [177]. In another case, *B. coagulans* GKN316, developed through atmospheric and room temperature plasma mutagenesis, exhibited strong resistance to a range of pretreatment-derived inhibitors [178].

Enhancing thermotolerance in probiotic strains is another valuable improvement for saline/marine plant-based biorefineries. Greater heat resistance can facilitate simultaneous saccharification and fermentation (SSF), reducing processing time, lowering enzyme demands, and improving strain survival during high-temperature steps like separation or drying [179–181]. For example, Prasad et al. [180] used ALE to develop thermotolerant *L. bulgaricus*, resulting in reduced enzyme usage and improved lactic acid yields during SSF. Additionally, ALE increased the viability of *L. paracasei* NFBC 338 by 18-fold compared to controls during spray drying at outlet temperatures of 95–105 °C [179]. Strain improvement, whether through genetic engineering or adaptive evolution, is essential for enhancing robustness, substrate utilization, and inhibitor tolerance in saline/marine biomass biorefineries. Continued development and scale-up of these optimized strains will be key to improving process efficiency and enabling sustainable, economically viable production systems.

### Future Outlook

Although high-salinity feedstocks can pose challenges for equipment maintenance and microbial conversions due to their inhibitory effects on microbial activity, they also serve as an effective contamination control mechanism, suppressing the growth of undesirable microorganisms and maintaining process stability [182]. Additionally, there is a growing trend toward utilizing saltwater for cultivation media, driven by concerns over freshwater depletion and cost efficiency, particularly in biofoundries with volumes of 1 million liters or more [183], overall fueling interest and investment in salt-involving processes.

Despite the promising potential of saline/marine plant-based probiotic production, several research gaps remain. Future studies should focus on:

1. Screening and characterization: identifying and analyzing a broader range of saline and marine plant species for their nutritional composition, prebiotic properties, and ability to support probiotic growth [45]
2. Optimal probiotic strains: selecting probiotic strains that efficiently utilize the nutrients in these plants and exhibit resilience to industrial processing conditions
3. Cost-effective bioconversion processes: developing innovative and economically viable bioconversion techniques to extract and process bioactive compounds, following biorefinery principles [184, 185]
4. Life cycle assessments: conducting comprehensive evaluations to assess the environmental and economic sustainability of saline/marine plant-based probiotic production [3]
5. Synergistic effects: investigating potential benefits of combining saline/marine plant extracts with other prebiotics or probiotics to enhance their efficacy [186]
6. Expanded applications: exploring novel applications of these probiotics in human and animal health beyond gut health, including immune support and metabolic health [187]

By addressing these gaps, the integration of saline and marine plants into probiotic production could become a sustainable and economically viable strategy, contributing to both the nutraceutical industry and environmental conservation efforts.

## Conclusion

In conclusion, the use of saline and marine feedstocks, such as seaweeds and halophytes, for the production of probiotics and postbiotics, presents significant opportunities across economic, environmental, and industrial domains. Probiotic strains, particularly those from *Lactobacillus* genera, as well as yeast species like *Saccharomyces* and *Candida*, are generally salt-tolerant and have demonstrated the ability to thrive in agal and halophytic environments, making them ideal candidates for fermentation in diverse conditions. These strains not only exhibit remarkable adaptability but are also known for their ability to produce bioactive compounds, such as antioxidants, phenolics, and antimicrobial agents, which can enhance the overall health benefits of fermented products and have promising applications in nutraceuticals and functional foods. Moreover, the use of mixed bacterial and fungal cultures in fermentation can enhance the diversity and robustness of the microbial ecosystem, improving the stability and functionality of the final product.

It has been shown that the inherent presence of antimicrobial compounds in algal and halophytic species further amplifies the growth of probiotics while providing effective pathogen and contamination control during the fermentation process, thus reducing the need for expensive sterilization steps and minimizing the use of preservatives. This synergy creates a safer and more controlled fermentation environment, leading to products with improved nutritional value, enhanced shelf life, and greater consumer appeal.

From the perspective of circular economy, utilizing saline and marine plants as substrates for probiotic production could reduce media costs and environmental impact, as these plants do not require arable land or freshwater. The cultivation of seaweed and halophytes also contributes to carbon sequestration, aiding in climate change mitigation and ecosystem restoration. Furthermore, the development of integrated biorefinery facilities close to coastal regions could drive down operational costs, enhancing the economic feasibility of these processes. The production of value-added byproducts, such as plant bioactive extracts, could further bolster the profitability of saline plant-based probiotic systems. While challenges related to optimizing fermentation conditions and improving scalability remain, the potential for marine and saline-based biorefineries to create sustainable, cost-effective probiotic and postbiotic products offers a promising pathway for both industrial innovation and environmental sustainability in the growing probiotic and nutraceutical market.

## Data Availability

No datasets were generated or analysed during the current study.
